# Influence of burnout and sleep difficulties on the quality of life among medical students

**DOI:** 10.1186/s40064-015-1477-6

**Published:** 2015-11-05

**Authors:** Daniel Pagnin, Valéria de Queiroz

**Affiliations:** Department of Psychiatry and Mental Health, Fluminense Federal University, Niterói, Marques do Paraná, 303 Prédio Anexo, Niterói, Rio de Janeiro, CEP 24030-215 Brazil

**Keywords:** Burnout, Sleep, Quality of life, Medical students, Medical education

## Abstract

This study assessed the influence of burnout dimensions and sleep difficulties on the quality of life among preclinical-phase medical school students. Data were collected from 193 students through their completion of the World Health Organization Quality of Life Instrument, the Maslach Burnout Inventory—Student Survey, the Mini-Sleep Questionnaire, the Social Readjustment Rating Scale, and the Beck Depression Inventory. This survey performed hierarchical multiple regressions to quantify the effects of emotional exhaustion, cynicism, academic efficacy, and sleep difficulties on the physical, psychological, social, and environmental components of an individual’s quality of life. The influence of confounding variables, such as gender, stress load, and depressive symptoms, were controlled in the statistical analyses. Physical health decreased when emotional exhaustion and sleep difficulties increased. Psychological well-being also decreased when cynicism and sleep difficulties increased. Burnout and sleep difficulties together explained 22 and 21 % of the variance in the physical and psychological well-being, respectively. On the other hand, physical health, psychological well-being, and social relationships increased when the sense of academic efficacy increased. Physical and psychological well-being are negatively associated with emotional exhaustion, cynicism, and sleep difficulties in students in the early phase of medical school. To improve the quality of life of these students, a significant effort should be directed towards burnout and sleep difficulties.

## Background

Quality of life is a subjective state of well-being, which can be influenced by culture, value systems, and stressful environments (The WHOQOL Group 1998). A university can be one of these stressful environments, despite its benefits to students. Non-medical and medical university students may experience a lower quality of life than young people in the general population (Dyrbye et al. 2006a; Henning et al. 2012).

Medical students endure a stressful learning environment, one that involves dealing with an overload of classes, patient diseases, and conflicting relationships with staff members (Dyrbye et al. 2009). Furthermore, specific milestones, such as the beginning of the clinical phase (Zhang et al. 2012), graduation (Willcock et al. 2004), internship, and residency training (West et al. 2011), are generally considered to be stressful transitions in medical education. Regardless of these later stressful transitions in the students’ education, some studies found earlier onset of burnout and sleep difficulties in the preclinical phase of medical school (Dyrbye et al. 2006a; Mazurkiewicz et al. 2012; Santen et al. 2010).

Burnout and sleep difficulties are related to distress in the learning environment during preclinical years (Mazurkiewicz et al. 2012). This student distress can lead to emotional exhaustion, depersonalization and reduced personal accomplishment, which are burnout dimensions according to Maslach et al. (1996). When adjusted to students, burnout concept comprises emotional exhaustion, cynicism, and low academic efficacy. Student distress can also lead to sleep difficulties, which include problems in falling asleep, mid-sleep awakenings, waking up too early, snoring, excessive daytime sleepiness, and restless sleep (Falavigna et al. 2011).

Burnout and sleep disorders are interconnected problems (Pagnin et al. 2014; Jansson-Frojmark and Lindblom 2010) with prevalence rates as high as 71 % (Mazurkiewicz et al. 2012) and 61 % (Rodrigues et al. 2002), respectively, in medical students. The exhausted students, who are overloaded with academic demands, typically use such inadequate coping mechanisms (such as sleep deprivation) to extend their study time. Thus, by sleeping less, these students become more exhausted. As a result, a chronic circle develops with high levels of burnout and sleep difficulties reinforcing each other.

In addition, burnout and sleep disorders can lead to serious consequences like anxiety disorders, depression (Dyrbye et al. 2006b), drug abuse, suicidal thoughts (Dyrbye et al. 2008), dropping out (Dyrbye et al. 2010b), decreased empathy (Thomas et al. 2007), low motivation for learning (Carskadon 2011), and poor academic performance (Rodrigues et al. 2002; Abdulghani et al. 2012). As such, burnout and sleep difficulties may affect the quality of life of medical students (Henning et al. 2009).

Nevertheless, little attention has been paid to how the combination of burnout and sleep difficulties affect the quality of life of medical students. In an attempt to assess how quality of life can be affected by student distress, our study is aimed to quantify the influence of burnout and sleep difficulties on the quality of life of medical students in the preclinical phase.

## Methods

This study assessed the influence of burnout and sleep difficulties on one’s quality of life while controlling the effects of gender, stress with life events, and depressive symptoms. The target population was second-year medical students who were in the last semester of the preclinical phase at the Fluminense Federal University (Niterói, Brazil). After about 30 days into the semester, we invited second-year medical students to complete self-administered questionnaires at the end of a lecture. The data sample was obtained from three different classes during three consecutive semesters. Students participated voluntarily, and did not receive any incentive. This study was approved by the local research ethics committee and all participants signed informed consent forms for the research study.

The standardized self-report questionnaires were the short version of World Health Organization Quality of Life Instrument, the Maslach Burnout Inventory—Student Survey, the Mini-Sleep Questionnaire, the Social Readjustment Rating Scale, and the Beck Depression Inventory.

The World Health Organization Quality of Life Instrument (WHOQOL-BREF) is a self-assessment questionnaire which measures individual perception of quality of life in the last 2 weeks (The WHOQOL Group 1998). It is comprised of 26 questions, with responses from 1 to 5 on a Likert scale in a positive direction except for three negatively worded items. The first two items separately assess the overall perception of quality of life and health. The remaining 24 items are divided into the four domains of physical, psychological, social relationships, and environment. The domain scores are calculated from the mean scores of respective items and these can be converted to a 0–100 scale. Higher scores mean a higher quality of life. The WHOQOL-BREF has a good internal consistency, discriminant validity, criterion validity, concurrent validity, and test–retest reliability. According Fleck et al. (2000), the Cronbach’s alpha is 0.77 for the domains and 0.91 for the questions; and the correlation coefficients range from 0.69 to 0.8. In our study, the Cronbach’s alpha coefficients for physical, psychological, social relationships, and environment domains were 0.78, 0.78, 0.71, and 0.71, respectively.

The Maslach Burnout Inventory—Student Survey (MBI-SS) is an adapted version of the Maslach Burnout Inventory—General Survey (MBI-GS) that has been used among students in many countries (Fang et al. 2012; Kusurkar et al. 2011; Galan et al. 2011; Hu and Schaufeli 2009; Dias et al. 2012; Schaufeli et al. 2002). The MBI-SS is formulated to assess student burnout in the academic environment and addresses three subscales of burnout which are emotional exhaustion, cynicism, and academic efficacy. The five questionnaire items of emotional exhaustion refer to severe fatigue caused by academic demands; the four items of cynicism refer to the student’s mental distance from lessons; and the six items of academic efficacy refer to academic accomplishment. These questionnaire items are answered in terms of frequency on a seven-point Likert scale (0 = never, 1 = a few times a year, 2 = once a month or less, 3 = a few times a month, 4 = once a week, 5 = a few times a week, 6 = every day) (Maslach et al. 1996). Similar to the MBI-GS, the MBI-SS assesses burnout as a three-dimensional construct. The use of the MBI-SS as a unidimensional concept (total score) was not yet validated and, therefore, cutoff points of total score were not established. Thus, our study used the mean scores of each subscale as a continuous variable. Higher scores mean higher emotional exhaustion, cynicism and academic efficacy. In the three-dimensional burnout concept, high scores on emotional exhaustion and cynicism and low scores on academic efficacy (academic efficacy items are reverse scored) indicate a high degree of burnout. In college students, the MBI-SS results show a satisfactory internal consistency. The Cronbach’s alphas were 0.81, 0.59, and 0.74 in the dimensions of emotional exhaustion, cynicism, and academic efficacy, respectively (Carlotto and Câmara 2006). In the current study, the Cronbach’s alpha were 0.77, 0.80, and 0.77 in the dimensions of emotional exhaustion, cynicism, and academic efficacy, respectively.

The Mini-Sleep Questionnaire (MSQ) is a self-report of current sleep quality, which is measured by ten questions about the frequency of sleep difficulties. Answer options range from 1 (never) to 7 (always), with higher scores meaning more sleep difficulties. The total MSQ score is classified into good sleep (10–24), mild sleep difficulties (25–27), moderate difficulties (28–30), and severe difficulties (>30). The MSQ presents good internal consistency and a good reliability in the test–retest analysis. In undergraduate students, the Cronbach’s alpha was 0.77 and Pearson’s R ranged from 0.72 to 0.92, with exception one item (R = 0.55) (Falavigna et al. 2011). In our sample of medical students, we found a Cronbach’s alpha of 0.77.

The Social Readjustment Rating Scale (SRRS) covers 43 life events with potential stress loads. Notable events are changes in family, occupation, economics, residence, affective, and social relationships in the last 12 months. When the event’s presence is checked, it receives a specific score. The scores of checked items are summed and classified as being low (≤149), mild (150–199), moderate (200–299), and major (≥300) (Holmes and Rahe 1967). The SRSS’s reliability among medical students showed a high correlation (r = 0.93) (Mendels and Weinstein 1972) when compared with the findings of the original report (Holmes and Rahe 1967). After 1 year, the retest consistency of medical students also revealed a high correlation (r = 0.94) (Mendels and Weinstein 1972). Our survey assessed the Cronbach’s alpha based on standardized items, which was of 0.66.

The Beck Depression Inventory (BDI) assesses depressive symptoms in the last week. This scale comprises 21 self-report items and these responses are scored on a 0–3 scale describing severity levels (Beck et al. 1961). The total score ranges from 0 to 63 and usually scores from 0 to 9 can be interpreted as normal; 10–18 as mild/moderate level; 19–29 as moderate/severe; and 30–63 as severe (Beck et al. 1988; Gorenstein and Andrade 1996). In college students, the BDI maintained high reliability with a Cronbach’s alpha of 0.86 (Gorenstein et al. 1999). In medical students, our study found a Cronbach’s alpha of 0.87.

### Statistical analysis

To summarize the sample characteristics, we used descriptive statistics as frequency, relative frequency, mean, and standard deviation. To assess the influence of burnout and sleep difficulties on quality of life, we performed hierarchical multiple regressions controlling for the effects of gender, stress load, and depressive symptoms.

In these regression analyses, the mean scores of burnout subscales and the total score of sleep quality were the independent variables, whereas the scores of the domains of quality of life were the dependent variables. The covariates of gender, stress load, and depressive symptoms were inserted in the first blocks of regression analyses. To assess the assumption of multicollinearity, the correlations between the variables in the model were verified. Data were analyzed by the statistical package SPSS 17.0 for Windows. Missing data were replaced by the process of simple imputation.

## Results

### Sample characteristics

Of the target population, 193 students completed and returned the questionnaires which resulted in a response rate of 87.7 % (193/220). Preclinical medical students had a mean age of 21.42 (SD = 2.41).

Considering the controlled covariates, women predominated slightly (53.9 %, 104 of 193 respondents) in the survey sample. Moreover, this sample from the 193 respondents presented a mild/moderate level of depressive symptoms (M = 13.74, SD = 8.94) and a moderate load of stress with life events (M = 203.46, SD = 104.65). Specifically, 70 % (137/193) of the students reported minimal or mild/moderate depressive symptoms and 79.3 % (153/193) reported low, mild, or moderate stress with life events (Table 1).

**Table 1:** Severity levels of stress with life events, depressive symptoms, quality of life, burnout, and sleep quality in 193 medical students

	N	%
Stress with life events (SRRS)		
Low (≤149)	71	36.8
Mild (150–199)	34	17.6
Moderate (200–299)	48	24.9
Major (≥300)	40	20.7
Depressive symptoms (BDI)		
No depression—minimal (0–9)	76	39.4
Mild-moderate (10–18)	61	31.6
Moderate-severe (19–29)	43	22.3
Severe (≥30)	13	6.7
Low quality of life^a^ (WHOQOL-BREF)		
Physical health	54	27.9
Psychological	79	40.9
Social relationships	101	52.3
Environment	32	16.6
Burnout (MBI-SS)		
Emotional exhaustion ≥4	124	64.2
Cynicism ≥4	37	19.2
Academic efficacy <4	84	43.5
Sleep quality (MSQ)		
Good sleep quality (10–24)	59	30.6
Mild difficulties (25–27)	32	16.6
Moderate difficulties (28–30)	24	12.4
Severe difficulties (>30)	78	40.4

^a^Below 25th percentile of the normative scores of general people aged between 20 and 29 (Cruz et al. 2011)

Considering the independent variables, medical students were shown to be emotionally exhausted and suffering from sleep difficulties (Table 1). The mean score of emotional exhaustion was 4.24 (SD = 1.05) and almost two-thirds of the students presented a score greater than or equal to 4, which meant that they felt emotionally exhausted at least once a week. Cynicism rates were lower than the emotional exhaustion. The mean score of cynicism was 2.21 (SD = 1.56) and only one-fifth of the medical students described detached attitudes at least once a week. The mean score of academic efficacy, which is a reverse subscale, was 3.98 (SD = 1.02) with more than half of the students feeling accomplished academically. Finally, the mean score of sleep quality was 29.09 (SD = 8.83), which showed a moderate level of sleep difficulties. In addition, 40.4 % (78/193) of the students showed severe sleep difficulties.

The quality of life measured by WHOQOL-BREF showed that the medical students suffered from a low quality of life. In the physical domain, the mean score was 59.55 (SD = 16.48); in the psychological domain, the mean score was 62.36 (SD = 16.01); in the social relationships domain, the mean score was 66.97 (SD = 20.51); and in the environment domain, the mean score was 61.97 (SD = 13.86). Compared to the general population (Cruz et al. 2011), a quarter to half of the students demonstrated low quality of life in the domains of physical health, psychological health, and social relationships. Furthermore, a sixth of the students had low quality of life in the environment domain (Table 1).

### Burnout and sleep quality as independent variables of quality of life

Table 2 details the associations between the quality of life domains and (1) the independent variables: burnout dimensions and sleep difficulties, and (2) covariates: stress with life events, depressive symptoms, and gender.

**Table 2:** Associations between quality of life domains and burnout dimensions, sleep quality, stress with life events, depressive symptoms, and gender

Variables	Physical health			Psychological			Social relationships			Environment		
	B (95 % CI)	T	p	B (95 % CI)	T	p	B (95 % CI)	t	p	B (95 % CI)	t	p
Emotional exhaustion	−3.69 (−5.77 to −1.61)	−3.49	*0.001*	−0.92 (−2.98 to 1.14)	−0.88	0.38	0.23 (−3.28 to 3.73)	0.13	0.90	−1.32 (−3.67 to 1.04)	−1.10	0.27
Cynicism	0.36 (−0.99 to 1.71)	0.52	0.61	−2.29 (−3.63 to −0.97)	−3.40	*0.001*	−2.09 (−4.36 to 0.19)	−1.81	0.07	−1.0 (−2.52 to 0.53)	−1.29	0.20
Academic efficacy	2.47 (0.64–4.29)	2.67	*0.008*	2.84 (1.04–4.64)	3.11	*0.002*	4.13 (1.06–7.21)	2.65	*0.009*	0.65 (−1.42 to 2.71)	0.62	0.54
MSQ	−0.60 (−0.82 to −0.38)	−5.32	*<0.001*	−0.35 (−0.57 to −0.13)	−3.15	*0.002*	−0.09 (−0.46 to 0.29)	−0.45	0.65	−0.14 (−0.39 to 0.12)	−1.06	0.29
SRRS	−0.01 (−0.02 to 0.01)	−0.27	0.79	−0.006 (−0.02 to 0.01)	−0.72	0.5	−0.002 (−0.03 to 0.02)	−0.13	0.90	−0.02 (−0.03 to 0.001)	−1.88	0.06
BDI	−0.49 (−0.69 to −0.29	−4.85	0.22	−0.64 (−0.84 to −0.45)	−6.43	*<0.001*	−0.51 (−0.85 to −0.17)	−2.99	*0.003*	−0.38 (−0.61 to −0.15)	−3.31	0.001
Gender	3.72 (0.25–7.19)	2.11	*0.04*	0.38 (−3.05 to 3.80)	0.22	0.83	−2.91 (−8.75 to 2.93)	−0.98	0.33	−1.41 (−5.33 to 2.51)	−0.71	0.48

Statistical significant associations in italic. *MSQ* Mini-Sleep Questionnaire, *SRRS* Social Readjustment Rating Scale, *BDI* Beck Depression Inventory

The medical students revealed a worse physical health when emotional exhaustion and sleep difficulties increased. For each one point increase in mean score of emotional exhaustion (MBI-SS), the score of physical health (WHOQOL-BREF) decreased by 3.7 points when the other variables are constant. Similarly, for one unit increase in MSQ score, physical health (WHOQOL-BREF) decreased by 0.6 units. In the psychological domain, the students had a poorer quality of life as cynicism and sleep difficulties increased. An increase in cynicism (MBI-SS) and MSQ scores of one unit was associated with a decrease in the psychological domain (WHOQOL-BREF) of 2.3 units and 0.4 units, respectively.

On the other hand, the academic efficacy subscale had a protective role. The more the sense of academic efficacy increased, the more the psychological well-being increased. One unit increase in academic efficacy (MBI-SS) was associated with an increase in the psychological domain (WHOQOL-BREF) of 2.8 units. Similarly, a greater sense of academic accomplishment correlated with better physical health and social relationships. One point increase in academic efficacy (MBI-SS) was associated with 2.5 and 4.1 points increase in physical health and social relationships (WHOQOL-BREF), respectively.

The burnout and sleep difficulties model was relevant in explaining quality of life findings after controlling for the effects of gender, stress load, and depressive symptoms. The physical and psychological domains were the most affected by burnout and sleep difficulties. Combining burnout dimensions and sleep difficulties explained 22 and 21 %, respectively, of the variance in the physical [R^2^ = 0.22, F(4, 185) = 23.04, p < 0.001] and psychological [R^2^ = 0.21, F(4, 185) = 21.07, p < 0.001] domains of quality of life. Moreover, burnout dimensions and sleep difficulties also explained 9 %, and 6 % of the variance in the social [R^2^ = 0.09, F(4, 185) = 5.21, p = 0.001] and environment [R^2^ = 0.06, F(4, 185) = 3.27, p = 0.01] domains.

### Covariates

Considering the covariates, gender and depressive symptoms were associated with quality of life. The male students presented better physical health than females. As expected, the increase of depressive symptoms was related to the worsening of psychological, social, and environmental domains (Table 2).

## Discussion

Burnout dimensions and sleep difficulties were related to a decrease of psychological well-being and physical health among preclinical medical students. In the physical health domain, the students scored worse when emotional exhaustion and sleep difficulties increased. In the psychological domain, the score decrease was associated with the score increase in the cynicism and sleep difficulties. On the contrary, the psychological well-being, physical health, and the social relationships were better when the sense of academic efficacy increased.

The quality of life in medical students is considered to be affected by burnout and sleep difficulties; however, the quantitative impact is unknown. Studies have addressed the relationship between burnout and sleep (Mazurkiewicz et al. 2012) or between burnout and quality of life (Henning et al. 2009) among medical students. In a different way, our study appraised the combined effect of burnout and sleep difficulties on quality of life, measuring quantitatively the influence of both factors in preclinical medical students. The quality of life presented a compelling association with burnout dimensions and sleep difficulties. Together, they explained 22 and 21 % of the variance in the physical and psychological well-being, respectively.

Quality of life is directly linked to health care. Although medical students learn how to promote health care in patients they sometimes neglect their own health care. In this incongruous situation, medical students may associate health care with the weakness of a sick person, weaknesses which they deny as being relevant to their own physical and mental well-being. In addition, some medical academic learning environments can promote feelings of omnipotence in which future doctors feel that they have to be able to endure extreme academic demands and distressing situations. Thus, these distressing situations can promote sleep difficulties, cynicism, emotional exhaustion, and, consequently, a low quality of life in medical students.

In the medical students surveyed, the decrease in physical well-being was connected with emotional exhaustion and low sleep quality. The physical domain of the WHOQOL-BREF includes activities of daily life and work capacity, which depend on the restorative functions of the body. These functions are affected by emotional exhaustion, which in turn reduces one’s energy and engagement with academic demands. Moreover, the sleep difficulties affect the vitality for everyday work and intensify sensitivity to the stressful experiences of medical learning (Vandekerckhove and Cluydts 2010; Zohar et al. 2005).

In our study, psychological well-being related negatively to cynicism and sleep difficulties. Students with detached attitudes toward medical learning are less likely to maintain altruistic and positive attitudes regarding social responsibility (Dyrbye et al. 2010a). This detached attitude can explain the presence of negative feelings, low self-esteem, and disbeliefs in the psychological domain of the WHOQOL-BREF. Furthermore, the sleep difficulties may have affected other items of the psychological domain such as memory and concentration.

On the other hand, in our survey psychological well-being, physical health, and social relationships related positively to the sense of academic efficacy. The students’ sense of academic efficacy may affect self-esteem, positive feelings, personal health, and learning ability. More confident students can express their knowledge and doubts because they are involved with the learning process. Consequently, they interact better with peers and professors. Nevertheless, a mutual positive reinforcement may occur between academic efficacy and quality of life. Medical students with better psychological and physical health can better handle the problems they face in the academic learning.

Educators and academic leaders have traditionally limited their role to academic teaching, and do not consider the effects of the learning environment on the physical and psychological well-being. Our study highlights the impact of medical school on quality of life through two negative effects, that is, burnout and sleep difficulties. Thus, our study suggests that educators and academic leaders should encourage stress management programs and sleep education in order to improve quality of life among students in the early phase of medical school.

A disadvantage regarding the study’s methodology was that the data were collected at three different times in a cross-sectional manner. Furthermore, although this study controlled for the effects of some moderator variables, there may be other mediator and moderator variables that affected our results. Thus, future work should focus on identifying the long-term effects of burnout and sleep difficulties on the quality of life among medical students and doctors. Moreover, other confounders, such as cognitive abilities and personality traits, should be explored to clarify their role.

## Conclusions

Our results highlight the influence of burnout and sleep difficulties on the quality of life among preclinical medical students. In this study, physical health and psychological well-being were found to be affected by burnout and sleep difficulties. Thus, in order to improve quality of life in the early phase of medical school students, strategies need to be created to manage stress within the learning environment, to inform students of the importance of adequate sleep, and to strengthen academic efficacy.
